# Supplementary material for: CD276-CAR T cells and Dual-CAR T cells targeting CD276/FGFR4 promote rhabdomyosarcoma clearance in orthotopic mouse models
Source: J Exp Clin Cancer Res. 2023 Nov 4;42:293. doi: 10.1186/s13046-023-02838-3 (PMC10625270; doi:10.1186/s13046-023-02838-3)
Supplement: Supplementary file 1 — Additional file 1: Supplementary Table S1. Primers used in this study. Supplementary Table S2. sgRNA sequences used to target CD276 and FGFR4. Supplementary Table S3. List of the CAR plasmids used for the production of CAR T cells. Supplementary Table S4. Statistical analysis of sdAb-FR4-CAR T cells killing capacity. Supplementary Table S5. T cells infection efficiency assessed at day 6 by GFP and CAR expression assessed by MycTag expression. Supplementary Table S6. Mean CD4+:CD8+ ratio at day 14 of production. Supplementary Table S7. Mean CAR expression assessed at d14 by GFP and MycTag expression. Supplementary Table S8. GFP and MycTag expression in one representative donor. Supplementary Table S9. Killing capacity significance compared to UTD T cells. Supplementary Table S10. Summary of memory phenotype measurements for three donors. Supplementary Table S11. Summary of exhaustion phenotype measurements for three donors. Supplementary Fig. S1. Validation of FGFR4 KO Rh4 cell line. Supplementary Fig. S2. Expression levels of the different CARs on Jurkat T cells. Supplementary Fig. S3. Activation profile of T cells and lentiviral infection efficiency. Supplementary Fig. S4. Evaluation of killing capacity by CD276-, F8-FR4-, and CD19-directed CAR T cells after co-incubation with RD, Rh4 and JR cell lines. Supplementary Fig. S5. Cytokine release by CD19-CAR T cells after 24h co-incubation with RD and Rh4 cells. Supplementary Fig. S6. Phenotypic characterization of CAR T cells on day 7 and day 14 before co-incubation. Supplementary Fig. S7. Phenotypic characterization of CAR T cells on day 16 after co-incubation with Rh4 and JR cells. Supplementary Fig. S8. Phenotypic characterization of CAR T cells during expansion and co-incubation experiments. Supplementary Fig. S9. No visible toxicity detected in normal mouse tissues by IHC. [file 13046_2023_2838_MOESM1_ESM.docx]

**Additional Files**

**Supplementary Tables:**

# Supplementary Table S1. Primers used in this study.

| Primer name | Sequence |
| --- | --- |
| P2A-tCD19 Forward | CGGCGCCACCAACTTCAGCCTGCTGAAGCAGGCCGGCGACGTGGAGGAGAACCCCGGCCCCCCACCTCCTCGCCTCCTC |
| P2A-tCD19 Reverse | TTCACAAATTTTGTAATCCAGAGGTTGATTGTCGACTTAATCACAGGACCAGGGCTCTT |

# Supplementary Table S2. sgRNA sequences used to target CD276 and FGFR4.

| Direction | Target | Guide Sequence (5' -> 3') | PAM | Exon |
| --- | --- | --- | --- | --- |
| + | CD276 | GCTGGTGCACAGCTTTGCTG | AGG | 8 |
| + | FGFR4 | TGGTGGCCACTGGTACAAGG | AGG | 6 |

# Supplementary Table S3. List of the CAR plasmids used for the production of CAR T cells.

| Plasmid | CAR | Addgene # |
| --- | --- | --- |
| MB0106 | CD19.8h.8TM.28.3z | 200670 |
| MB0107 | CD19.8h.8TM.BB.3z | 200671 |
| MB0108 | CD19.8h.8TM.28.BB.3z | 200672 |
| MB0109 | CD19.8h.8TM.BB.28.3z | 200673 |
| MB0110* | CD19.8h.28TM.28.3z | 200674 |
| MB0111* | CD19.8h.28TM.BB.3z | 200675 |
| MB0112* | CD19.8h.28TM.28.BB.3z | 200676 |
| MB0113* | CD19.8h.28TM.BB.28.3z | 200677 |
| MB0114 | CD19.28h.28TM.28.3z | 200678 |
| MB0115 | CD19.28h.28TM.BB.3z | 200679 |
| MB0116 | CD19.28h.28TM.28.BB.3z | 200680 |
| MB0117 | CD19.28h.28TM.BB.28.3z | 200681 |
| MB0118 | CD276.8h.8TM.28.3z |  |
| MB0119 | CD276.8h.8TM.BB.3z |  |
| MB0120 | CD276.8h.8TM.28.BB.3z |  |
| MB0121 | CD276.8h.8TM.BB.28.3z |  |
| MB0126 | CD276.28h.28TM.28.3z |  |
| MB0127 | CD276.28h.28TM.BB.3z |  |
| MB0128 | CD276.28h.28TM.28.BB.3z |  |
| MB0129 | CD276.28h.28TM.BB.28.3z |  |
| MB0130 | F8-FR4.8h.8TM.28.3z |  |
| MB0131 | F8-FR4.8h.8TM.BB.3z |  |
| MB0132 | F8-FR4.8h.8TM.28.BB.3z |  |
| MB0133 | F8-FR4.8h.8TM.BB.28.3z |  |
| MB0134 | F8-FR4.28h.28TM.28.3z |  |
| MB0135 | F8-FR4.28h.28TM.BB.3z |  |
| MB0136 | F8-FR4.28h.28TM.28.BB.3z |  |
| MB0137 | F8-FR4.28h.28TM.BB.28.3z |  |

*not used in this study

# Supplementary Table S4. Statistical analysis of sdAb-FR4-CAR T cells killing capacity.

The statistical significance of the differences between experimental and control groups was assessed using Dunnett’s multiple comparison test following a two-way ANOVA (p>0.05 (ns), p ≤ 0.05 (*), p ≤ 0.01 (**), p ≤ 0.001 (***), p ≤ 0.0001 (****)).

# Supplementary Table S5. T cells infection efficiency assessed at day 6 by GFP and CAR expression assessed by MycTag expression.

# Supplementary Table S6. Mean CD4^+^:CD8^+^ ratio at day 14 of production.

# Supplementary Table S7. Mean CAR expression assessed at d14 by GFP and MycTag expression.

# Supplementary Table S8. GFP and MycTag expression in one representative donor

# Supplementary Table S9. Killing capacity significance compared to UTD T cells.

The statistical significance of the differences between experimental and control groups was assessed using Dunnett’s multiple comparison test following a two-way ANOVA (p>0.05 (ns), p ≤ 0.05 (*), p ≤ 0.01 (**), p ≤ 0.001 (***), p ≤ 0.0001 (****)).

# Supplementary Table S10. Summary of memory phenotype measurements for three donors.

# Supplementary Table S11. Summary of exhaustion phenotype measurements for three donors.

**Supplementary Figures:**


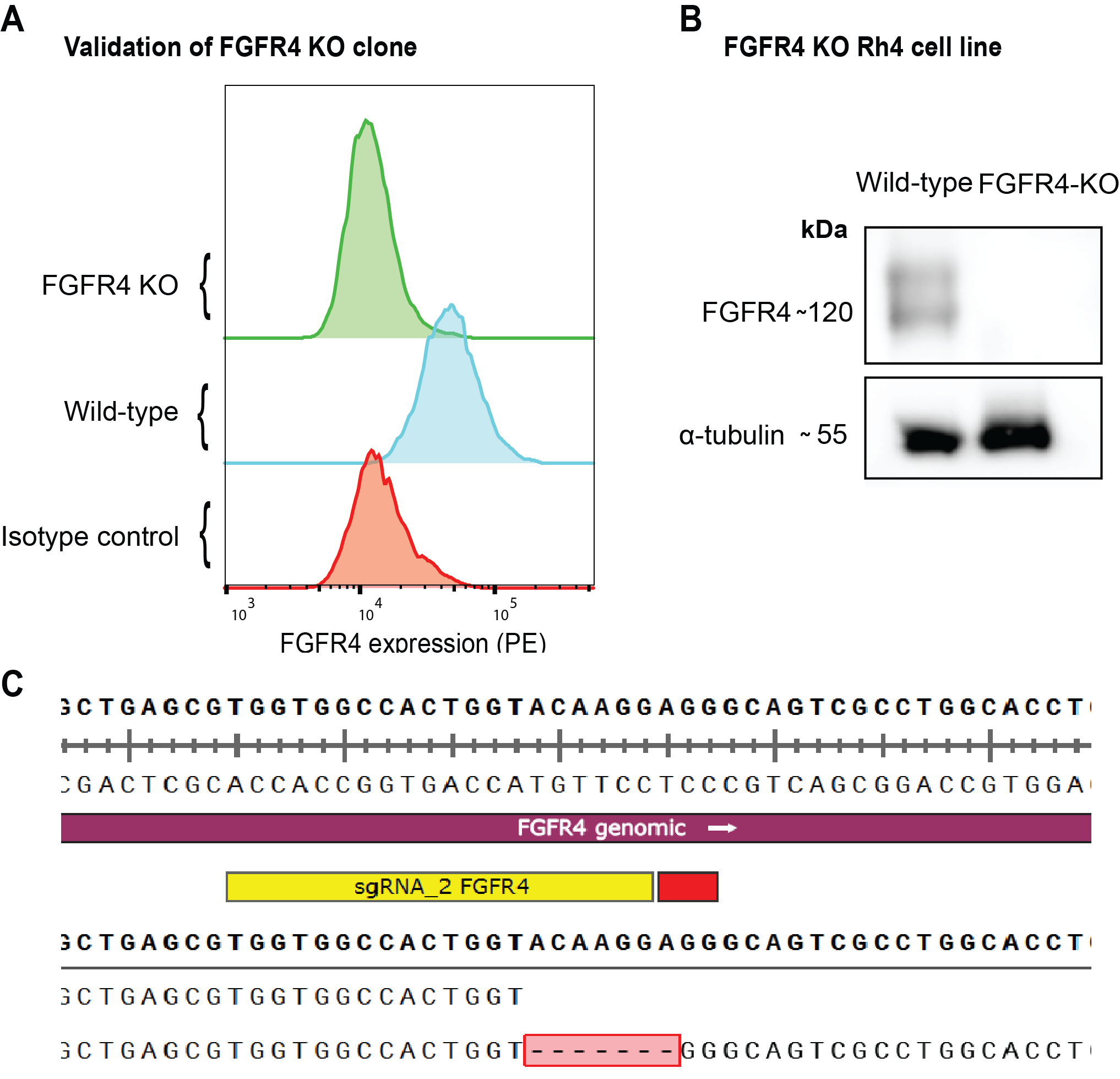


# Supplementary Fig. S1. Validation of FGFR4 KO Rh4 cell line.

(**A**) Flow Cytometry detection of FGFR4 in FGFR4 KO clone (green), compared to Rh4 wild-type (cyan) and isotype control (red). (**B**) FGFR4 expression levels were evaluated by WB. No detection of FGFR4 was visible in the second lane, corresponding to the FGFR4 KO clone. (**C**) Sequencing of PCR amplified fragments cloned by TOPO TA cloning confirmed the introduction of a 7 bp-gap with consequent frameshift mutation. This change in the sequence in exon 6 results in no expression of FGFR4.

**
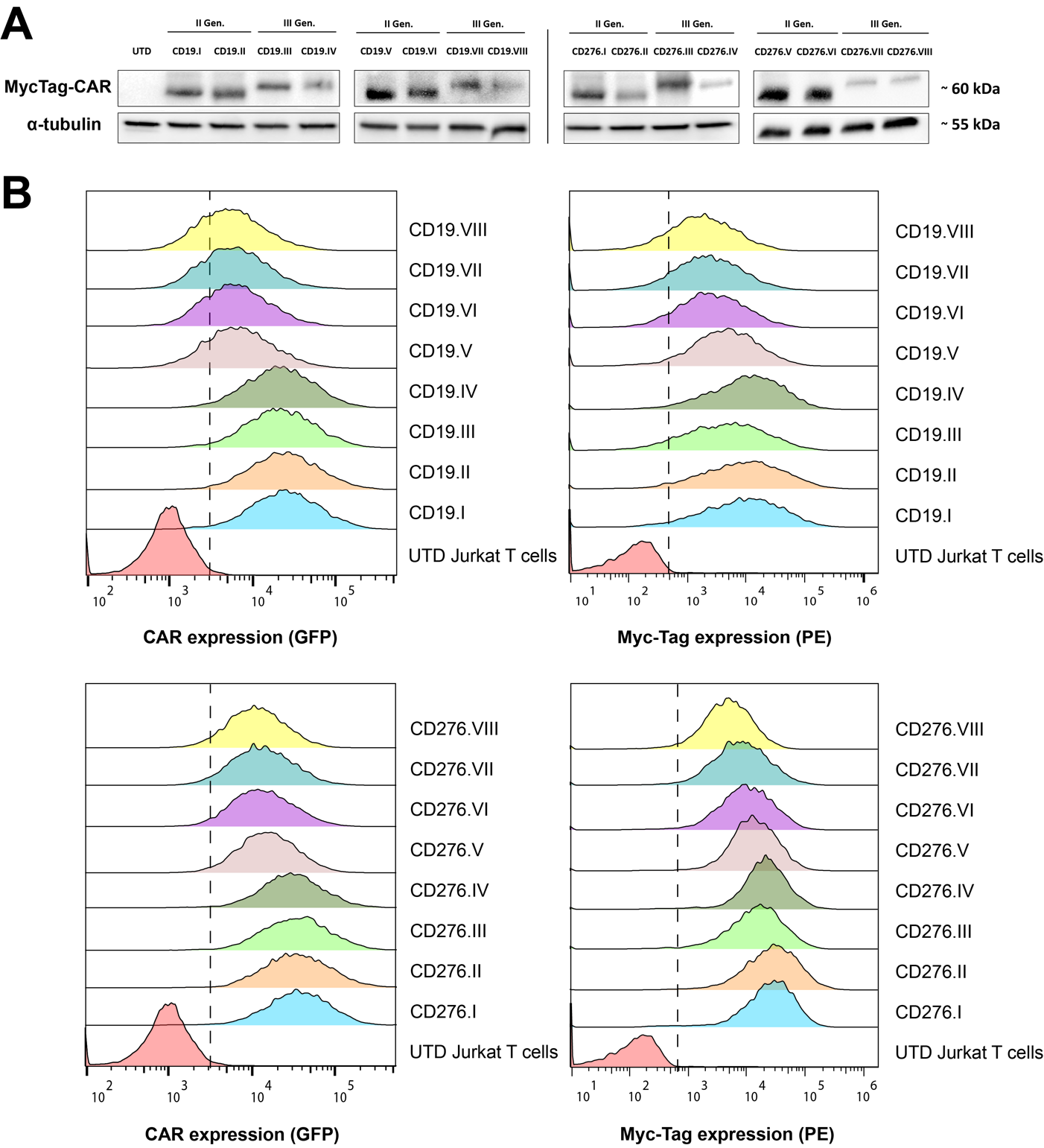
**

# Supplementary Fig. S2. Expression levels of the different CARs on Jurkat T cells.

(**A)** Western blotting analysis showed high CAR expression levels in all the constructs of interest by MycTag detection. The respective bands confirmed a predicted CAR size of ca. 60 kDa with a visible shift between the second- and third-generation CARs. (**B)** FACS analysis on living GFP-expressing CAR Jurkat T cells performed with an anti-MycTag PE-conjugated antibody confirmed high and homogenous surface expression of the CARs.

**
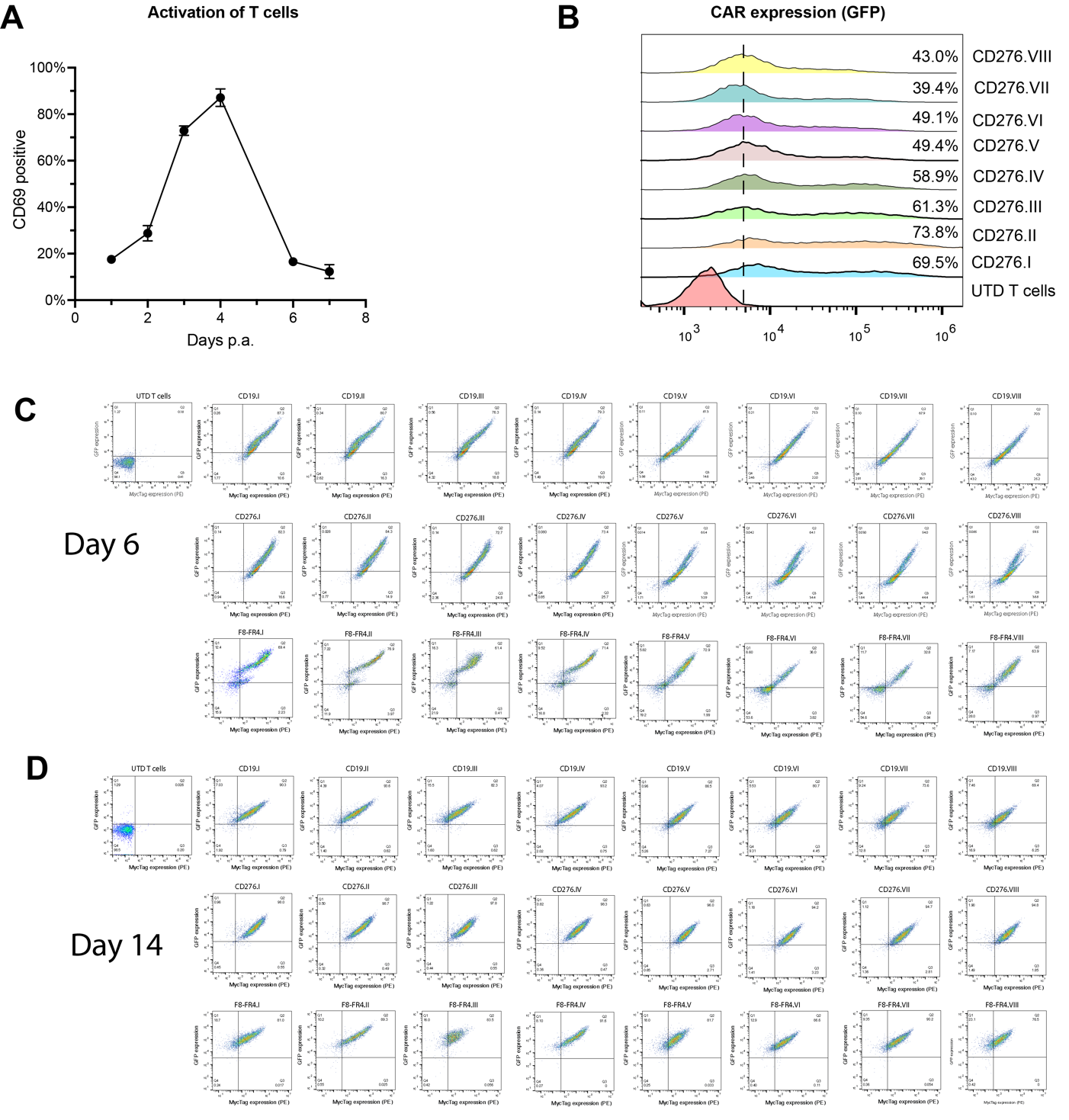
**

# Supplementary Fig. S3. Activation profile of T cells and lentiviral infection efficiency.

(**A**) T cells were purified from PBMCs and incubated on day 1 with anti-CD3/CD28 activators. During the initial T cell expansion, CD69 marker expression was monitored and resulted at the highest levels on day 4. (**B**) T cells were infected with CAR-expressing lentiviruses on day 4 and transduction efficiency was >40% in most of the tested CAR T cells. Infection efficiency and CAR expression efficiency monitored by GFP expression and MycTag expression, shown for one representative donor at Day 6 (**C**), and at day 14 (**D**).


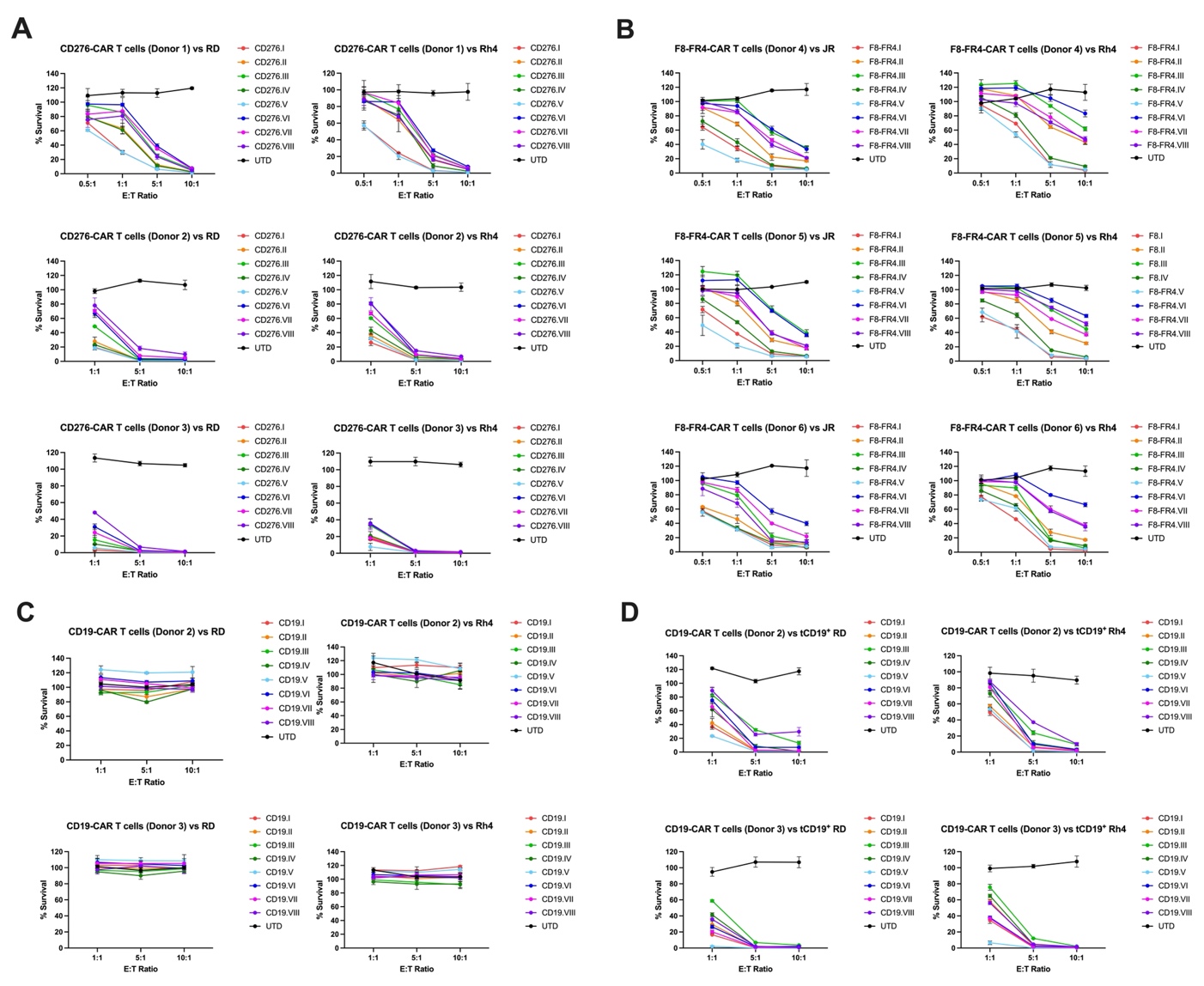


# Supplementary Fig. S4. Evaluation of killing capacity by CD276-, F8-FR4-, and CD19-directed CAR T cells after co-incubation with RD, Rh4 and JR cell lines.

(**A**) CD276-CAR T cells were co-incubated for 48h with fLuc^+^ RD and Rh4 at different E:T ratios showed potent killing of RMS cells in the three donors. CD276.V (cyan) was selected as the most potent CAR construct. (**B**) F8-FR4-CAR T cells from three different donors were co-incubated with fLuc^+^ JR and Rh4 showed high killing capacity at E:T ratios of 5:10 and 10:1. Similarly to the results observed with CD276 CAR T cells, F8-FR4.V CAR T cells outperformed the other experimental groups. (**C**) CD19-CAR T cells, used here as negative control, were co-incubated with fLuc^+^ RD and Rh4 and confirmed no unspecific killing of RMS cells. (**D**) CD19-CAR T cells, used here as positive control, were co-incubated with tCD19^+^ fLuc^+^ RD and Rh4. High killing efficacy was confirmed for all CD19-CAR constructs.

**
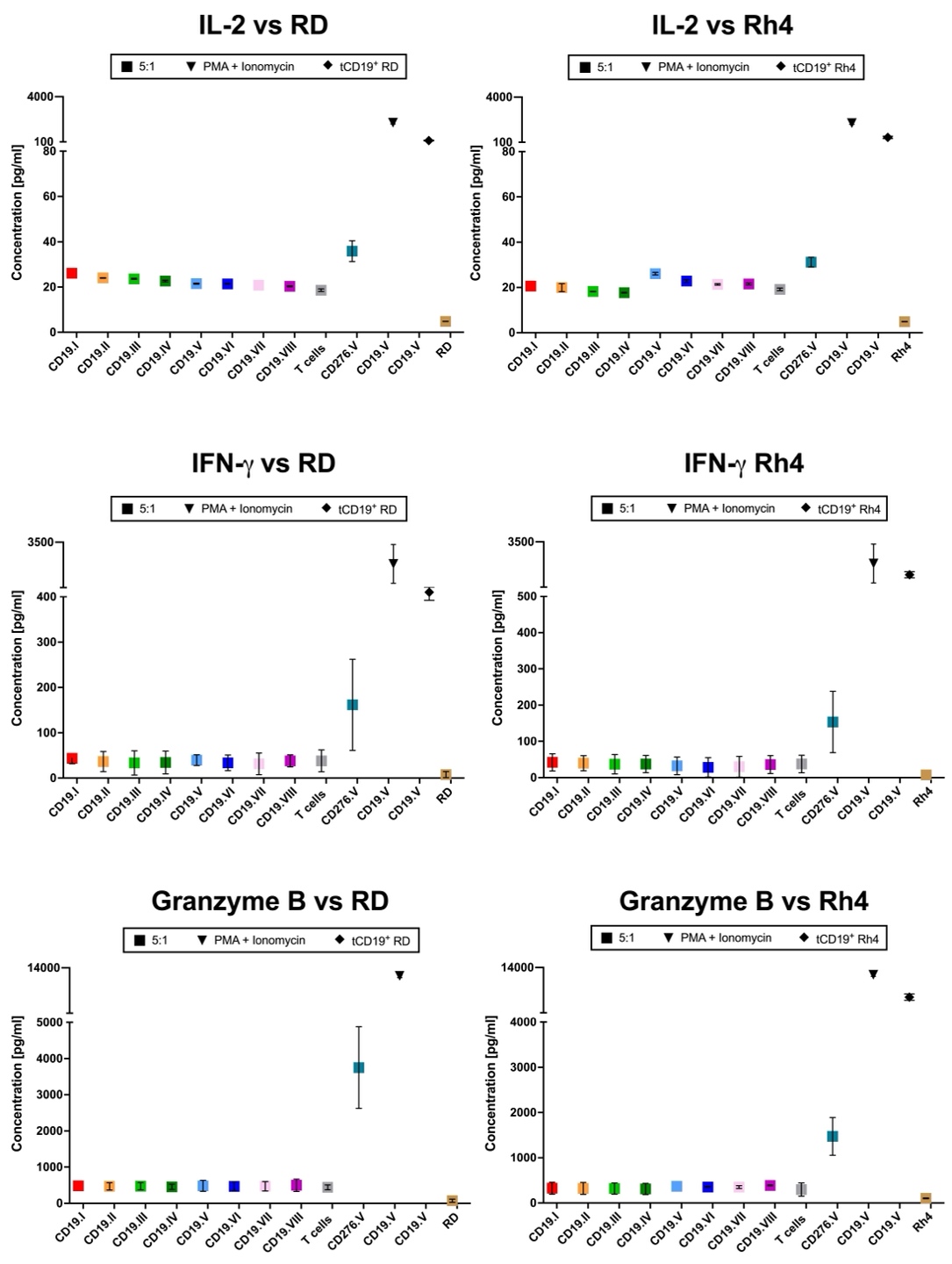
**

# Supplementary Fig. S5. Cytokine release by CD19-CAR T cells after 24h co-incubation with RD and Rh4 cells.

Concentrations of IL-2, IFN-γ, and Granzyme B in the supernatant released during co-incubation of CD19-CAR T cells with RD (left panels) and Rh4 cells (right panels) at the E:T ratio of 5:1 were measured by ELISA after 24h. For CD19-CAR constructs background levels of cytokines were detected. As positive control CD276.V-CAR T cells were used, as well as CD19.V-CAR T cells were also incubated with RD and Rh4 cells overexpressing a truncated version of CD19 (tCD19).


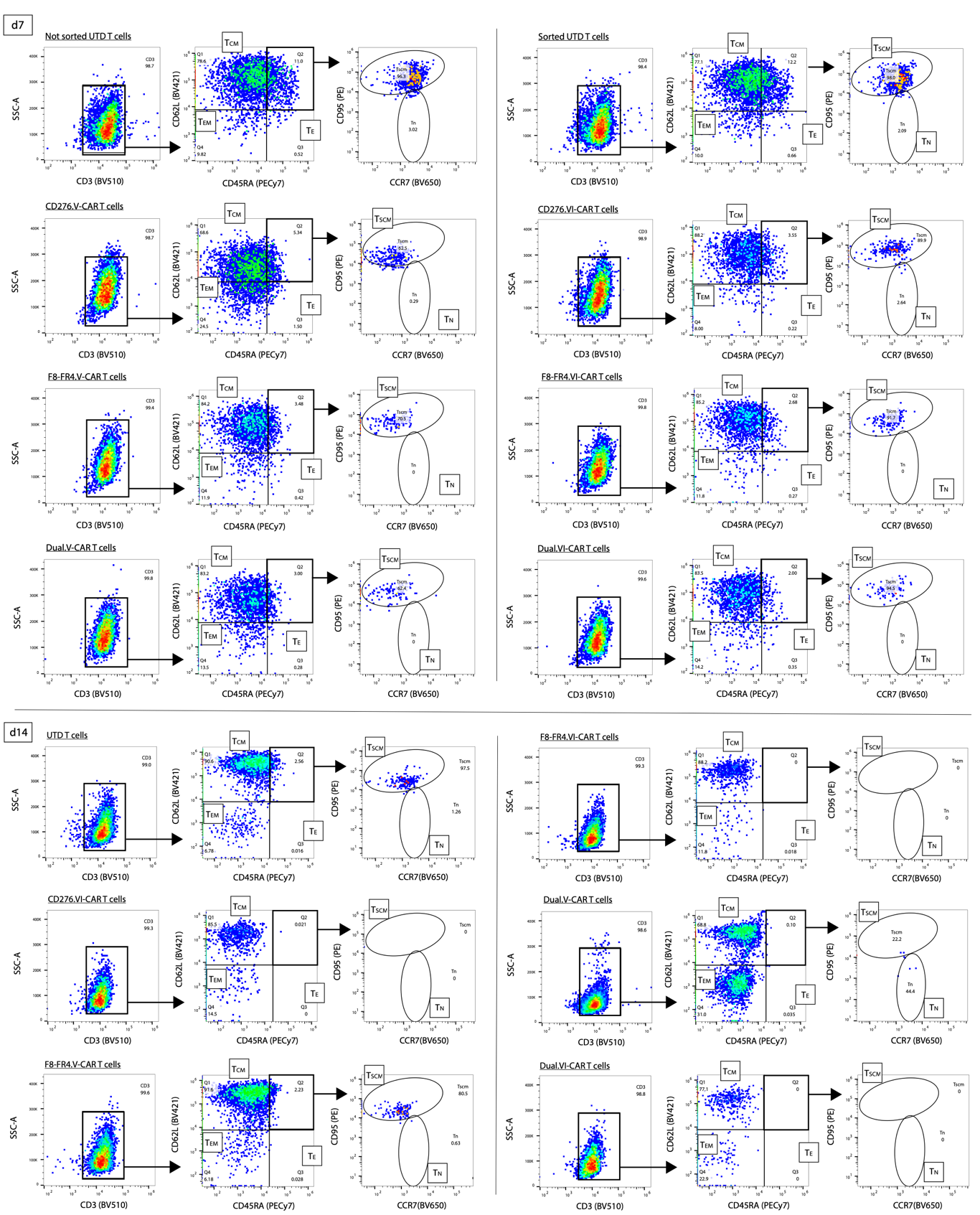


# Supplementary Fig. S6. Phenotypic characterization of CAR T cells on day 7 and day 14 before co-incubation.

Flow Cytometry during CAR T cells manufacturing was used to quantify percentages of cell memory (TCM), effector memory (TEM) and effector (TE) T cells on CD3 positive cells by CD45RA and CD62L staining. Naïve (TN) and stem cell-like (TSCM) T cells were quantified on CD3^+^/CD45RA^+^/CD62L^+^ based on CD95 and CCR7 staining. On day 14, before co-incubation assays activated CAR T cells showed very high percentage of TCM cells and lower percentages of the other cell populations.


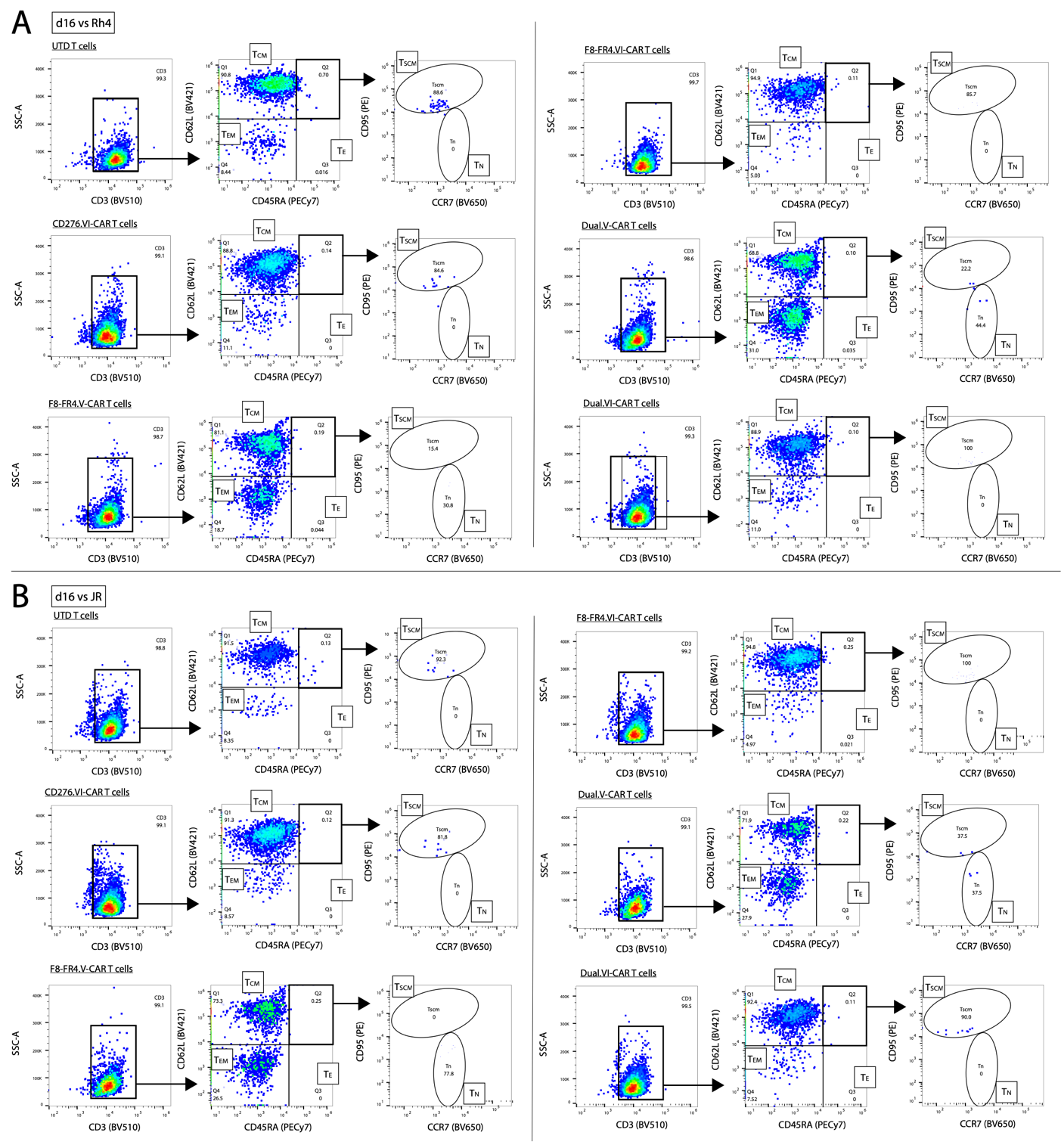


# Supplementary Fig. S7. Phenotypic characterization of CAR T cells on day 16 after co-incubation with Rh4 and JR cells.

Flow Cytometry during CAR T cells manufacturing was used to quantify percentages of cell memory (TCM), effector memory (TEM) and effector (TE) T cells on CD3 positive cells by CD45RA and CD62L staining. Naïve (TN) and stem cell-like (TSCM) T cells were quantified on CD3^+^/CD45RA^+^/CD62L^+^ based on CD95 and CCR7 staining. (**A**) On day 16, after co-incubation assays with Rh4 cells, almost 60% of activated CAR T cells had a TCM phenotype, and ca. 40% a TEM phenotype. (**B**) On day 16, after cytotoxicity experiments with JR, almost 70% were TCM cells, whereas ca. 30% were TEM cells.


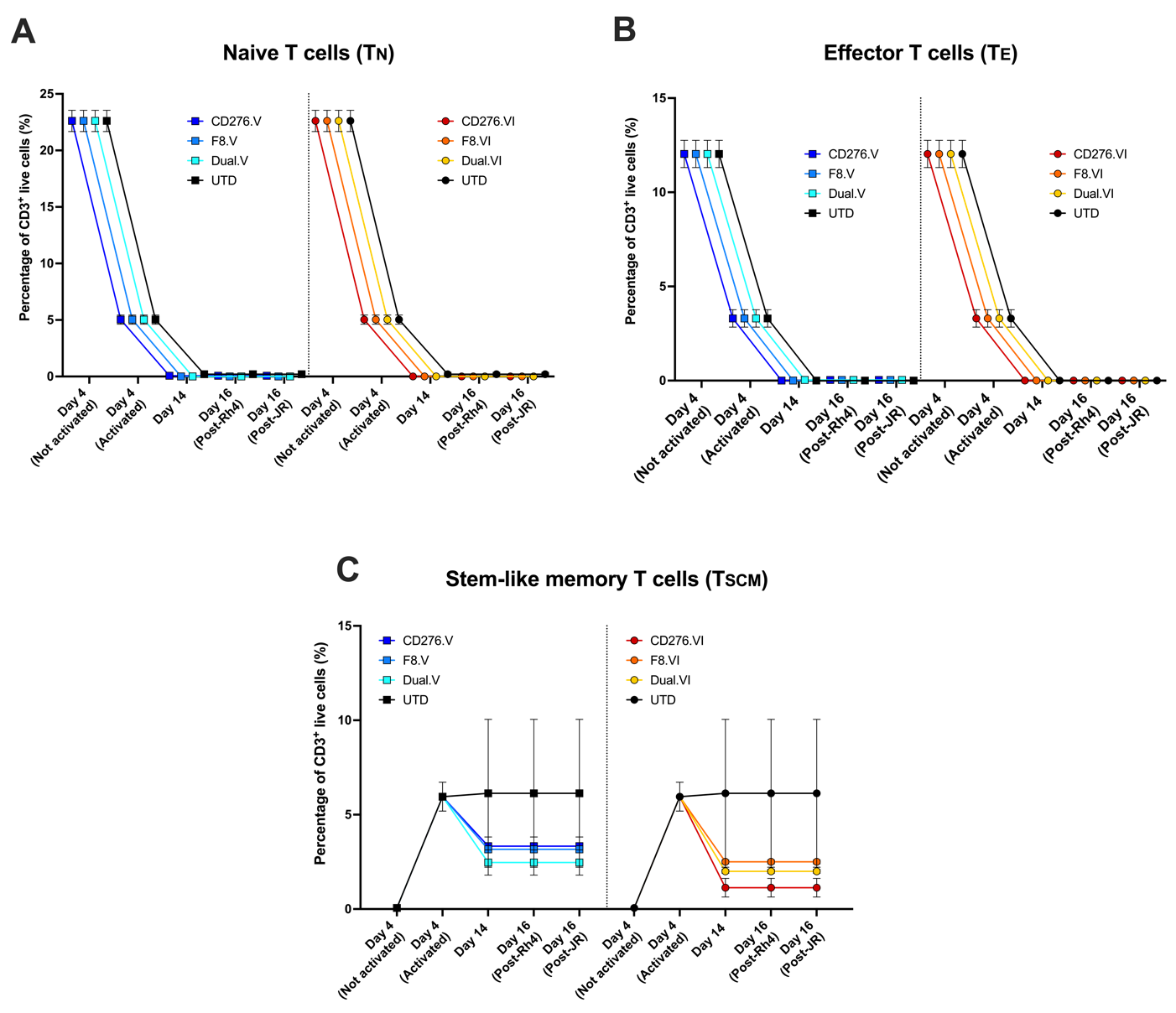


# Supplementary Fig. S8. Phenotypic characterization of CAR T cells during expansion and co-incubation experiments.

Flow Cytometry during CAR T cells manufacturing was used to quantify percentages of cell memory (TCM), effector memory (TEM) and effector (TE) T cells on CD3 positive cells by CD45RA and CD62L staining. Naïve (TN) and stem cell-like (TSCM) T cells were quantified on CD3^+^/CD45RA^+^/CD62L^+^ based on CD95 and CCR7 staining. (**A**) On day 4 before infection, almost 25% of not-activated T cells were TN. As expected, T cells activated for 4 days showed a lower TN population, and there were no naïve CAR T cells detected from day 14. (**B**) On day 4 before infection, less than 15% of not-activated T cells were TE. T cells activated for 4 days showed a lower TE population, and there were no effector CAR T cells detected from day 14. (**C**) ~5% of activated T cells were TSCM on day 4. Population decreased by ca. 2-fold during expansion and co-incubation experiments.

# Supplementary Fig. S9. No visible toxicity detected in normal mouse tissues by IHC.

IHC analyses performed on normal tissues show no apparent side effects due to the CAR T cell treatment. Hematoxylin and Eosin staining performed in untreated and CAR T cell-treated mice exhibited no evident side effects in normal tissues, such as liver, muscle, brain, heart, and spleen.
